# Novel Curcumin Floating Tablets for Spatial Delivery in Peptic Ulcer

**DOI:** 10.1155/bmri/6622146

**Published:** 2025-07-23

**Authors:** Chitra Gupta, Girdhar Kumar Sahu, Ashish Kumar Parashar, Kalpana Singh, Sarad Pawar Naik Bukke, Godswill James Udom

**Affiliations:** ^1^Department of Pharmaceutics, Lloyd Institute of Management and Technology, Greater Noida, 201306, India; ^2^Department of Pharmaceutics and Pharmaceutical Technology, Kampala International University, Western Campus, Ishaka-Bushenyi, PO Box 71, Uganda; ^3^Department of Pharmacology and Toxicology, Faculty of Pharmacy, Federal University Oye-Ekiti, Oye-Ekiti, Nigeria

**Keywords:** curcumin, cyclodextran complex, floating tablet, gastroretentive, *Helicobacter pylori*, peptic ulcers, solvent evaporation method

## Abstract

**Background:** Peptic ulcers, caused by factors like *Helicobacter pylori* infection and NSAID overuse, are often treated with drugs that can have significant downsides. Curcumin, a natural compound with high therapeutic potential, faces challenges due to poor solubility and instability. Innovative delivery systems are key to unlocking curcumin's full benefits for effective peptic ulcer treatment.

**Materials and Methods:** To address the challenge of curcumin's limited bioavailability, a novel drug delivery system was developed. The system employed a twofold strategy: enhancing curcumin's solubility via complexation with methyl-*β*-cyclodextrin, and formulating effervescent, gastroretentive tablets composed of hypromellose and gas-generating agents. This approach is aimed at achieving sustained gastric retention and localized drug release, thereby maximizing curcumin's therapeutic potential.

**Results:** The formulated tablets exhibited excellent pharmaceutical properties, meeting all required standards for hardness, friability, and drug content. Importantly, the tablets demonstrated rapid flotation in simulated gastric fluid (buoyancy achieved within 20 s) and maintained buoyancy for approximately 16 h, indicating successful gastroretention. *In vitro* dissolution studies confirmed sustained drug release (73.92% within 9 h) following a quasi-Fickian diffusion mechanism, as per the Korsmeyer–Peppas model.

**Conclusions:** This study presents a novel approach to enhance peptic ulcer treatment using floating tablets designed to optimize curcumin delivery. These tablets leverage cyclodextrin complexation for enhanced solubility, while their effervescent, gastroretentive properties allow for prolonged gastric residence time and targeted drug release. This strategy shows promise in improving curcumin's bioavailability and therapeutic efficacy, addressing a significant unmet need in peptic ulcer management.

## 1. Introduction

Peptic ulcers, a global health concern, involve erosion of the stomach or duodenal lining. These lesions can cause severe complications such as bleeding, perforation, and obstruction, significantly impairing a patient's quality of life [1]. Peptic ulcer development is influenced by a complex interplay of factors, including *Helicobacter pylori* infection, NSAID use, smoking, and genetic predisposition. *H. pylori* infection is a primary culprit, disrupting the delicate balance between aggressive factors (e.g., acid and pepsin) and protective mechanisms (e.g., mucosal defense) within the gastric mucosa [2, 3]. Historically, peptic ulcer treatment has centered on reducing stomach acid using H2 receptor antagonists and PPIs. Although effective in alleviating symptoms and promoting healing, the emergence of drug-resistant *H*. *pylori* and long-term NSAID side effects highlight the need for continued exploration of alternative treatment approaches [4].

Curcumin, a biologically active compound found in turmeric, is commonly consumed in daily diets, particularly in regions like India and South Asian Association for Regional Cooperation countries [5]. Chemically known as diferuloylmethane, with an IUPAC name of (1E-6E)-1,7-bis(4-hydroxy-3-methoxyphenyl)-1,6-heptadiene-3,5-dione and a molecular weight of 368.37 g/mol [6, 7]. Renowned for its diverse biological and pharmacological activities, curcumin has garnered significant attention in recent years [8, 9].

Curcumin's benefits extend to gastric health, where it aids in the prevention and alleviation of gastric lesions. It also exhibits antihypocholesterolemic, antiyeast, anticancer, antitumor-promoting, antimutagen, antiproliferative, and multidrug resistance (MDR) modulator effects [10]. Furthermore, it assists in the management of metabolic syndrome, anxiety, and hyperlipidemia, potentially alleviating exercise-induced inflammation and muscle soreness [11, 12].

The current work is aimed at exploiting curcumin's therapeutic potential in peptic ulcers by formulating floating effervescent tablets. Floating tablets offer a solution by prolonging the drug's stay in the stomach, thus improving localized release and action of the drug in peptic ulcers. However, curcumin's oral bioavailability is limited by its poor water solubility and instability at the alkaline environment of the intestinal [13]. This approach also addresses the issue of drug instability in the alkaline pH, as the tablets release the maximum drug while floating in the gastric environment [14]. This research is aimed at creating a buoyant drug delivery system utilizing hydroxypropyl methylcellulose (HPMC) to enhance the oral bioavailability of curcumin and prolong its residence in the stomach, potentially leading to prolonged absorption. To improve the solubility, curcumin was converted to its cyclodextran complex prior to incorporating into floating tablets. Through this research, we aim to advance therapeutic outcomes for curcumin in gastric ailments. Gastroretentive drug delivery systems (GRDDSs) prolong gastric residence time (GRT) and control drug release, making them popular as orally administered site-specific controlled release drug delivery systems [15]. Gastroretentive approach further solves the major challenges of variability in drug absorption among individuals due to physiological factors like gastrointestinal transit and GRT [16].

By prolonging the residence time of dosage forms in the gastric lumen, gastroretentive systems enhance drug bioavailability, minimize drug wastage, and improve the solubility of poorly soluble drugs in a high pH environment [17]. Several mechanisms, including floatation, mucoadhesion, swellable systems, and hydrodynamically balanced systems, have been used to achieve gastric retention [18].

Among various approaches to achieve gastric retention, floatation offers a simple yet effective strategy. Gastroretentive floating drug delivery systems, by virtue of their low bulk density relative to gastric fluids, remain buoyant in the stomach. This prolonged GRT allows for sustained drug release, ultimately enhancing drug absorption and bioavailability [19].

Floating drug delivery systems achieve prolonged GRT by remaining buoyant in the stomach, thereby enhancing drug bioavailability [20]. The duration of GRT, a key determinant of drug absorption, is influenced by a complex interplay of formulation parameters (e.g., dosage form size) and physiological factors (e.g., gastric pH and food intake) as well as patient-specific variables such as age, body mass index, and gender [21, 22]. Commonly employed gastroretentive dosage forms include floating beads, tablets, and microspheres, each offering distinct advantages and limitations depending on the desired drug release profile and therapeutic application [23].

Hydrophilic matrix systems, which utilize HPMC, are a practical approach for developing modified and sustained release dosage forms [24]. HPMC K15M may provide sustained and controlled release of curcumin by forming a gel barrier that regulates drug diffusion. Its high viscosity helps the tablet remain buoyant in gastric fluids, enhancing gastric retention. This may improve curcumin's bioavailability by prolonging its residence time at the absorption site. Matrix tablets manufactured via direct compression are cost-effective, offer flexibility in achieving desired release profiles and have broad regulatory acceptance [25]. In the present research work, we have developed floating tablets, designed to prolong GRT and facilitate controlled curcumin release, offering an encouraging avenue for targeted delivery of drug to the ulcer site [26].

## 2. Materials and Methodology

### 2.1. Materials

Curcumin (> 98% purity) was procured from Earth's Soul Ayurveda (Nature Code) Pvt. Ltd., located in Greater Noida, Uttar Pradesh, India. Methyl-*β*-cyclodextrin was purchased from Central Drug House, New Delhi. All remaining chemicals utilized were of AR grade and sourced from the college laboratory.

### 2.2. Methodology

#### 2.2.1. Preformulation Studies

Fourier transform infrared (FTIR) of pure curcumin was performed using ATR (Bruker) within the spectrum spanning from 4000 to 400 cm^−1^ [27]. Key absorption ranges included 1450–1600 cm^−1^ for C-C stretching in benzene rings, 1500–1680 cm^−1^ for C=C stretching in aromatic compounds, 1670–1750 cm^−1^ for C=O stretching in ketones, 3079–2920 cm^−1^ for stretching of C-H in aromatics, 3200–3500 cm^−1^ for O-H stretching with phenolics, and 1000–1300 cm^−1^ for C-O stretching in aromatics. Additionally, 1400–1500 cm^−1^ characterized C-H bending in olefins and 1050–1100 cm^−1^ for O-CH_3_ bending in methoxy groups (Senjoti et al. [28], Yamada et al. [29], and Kim et al. [30]).

The *λ*_max_ of curcumin was determined in 0.1 N HCl using a dual-beam UV-visible spectrophotometer (Shimadzu 1800 UV-Vis) [31]. Further, the calibration curve for curcumin was prepared using the UV-Vis spectrophotometric method. Serial dilutions of a 100-*μ*g/mL curcumin stock solution were prepared in 0.1 N HCl, yielding concentrations ranging from 10 to 70 *μ*g/mL. The absorbance of each solution was measured at 423 nm using a Shimadzu 1800 spectrophotometer. A calibration curve plotting absorbance against the corresponding curcumin concentration was generated, and linearity was assessed by linear regression analysis using Microsoft Excel [31, 32].

Solubility of the curcumin was determined by preparing a supersaturated solution of curcumin in 10-mL distilled water within a sealed volumetric flask. The flask was subjected to continuous agitation for 2 h at room temperature to ensure equilibrium saturation. The resulting supersaturated solution was then filtered through a 0.22-*μ*m filter to remove any undissolved curcumin particles. The filtrate was further diluted as needed, and its absorbance was measured at 423 nm using UV-Vis spectrophotometry [33].

#### 2.2.2. Assessment of Drug–Excipient Compatibility

FTIR spectroscopy was employed to assess the compatibility between curcumin and the selected excipients. Individual FTIR spectra were obtained for methyl-*β*-cyclodextrin, HPMC K15M, curcumin, a 1:1 curcumin-methyl-*β*-cyclodextrin complex, and a physical mixture of HPMC K15M with the aforementioned complex. Spectra were recorded using a Bruker FTIR instrument over a wavenumber range of 4000–400 cm^−1^. The resulting spectra were analyzed to identify any potential drug–excipient interactions as indicated by shifts in characteristic peaks or the emergence of new peaks [21].

#### 2.2.3. Preparation of Methyl-*β*-Cyclodextrin and Curcumin Inclusion Complexes

Methyl-*β*-cyclodextrin and curcumin were meticulously combined in equal proportions utilizing a mortar and pestle. Methanol was subsequently introduced in a sufficient amount to form a liquid. The liquid was left to evaporate at ambient temperature throughout the night. The resulting cyclodextrin complexes were then pulverized, subjected to drying in a hot air oven, and sieved through an 80-mesh sieve [33–35].

#### 2.2.4. Solubility of Curcumin–Cyclodextrin Complexes

The solubility of curcumin–cyclodextrin complexes was evaluated using a supersaturation method. An excess amount of the complex was added to 10 mL of distilled water in a sealed volumetric flask. Following the same protocol used for the curcumin solubility study, the mixture was agitated for a defined period to reach equilibrium. After filtration to remove any undissolved complex, the concentration of dissolved curcumin in the filtrate was determined, providing a measure of the complex's solubility [33].

#### 2.2.5. *In Vitro* Dissolution of Pure Curcumin and Inclusion Complexes

Dissolution profiles of pure curcumin and a 1:1 curcumin:methyl-*β*-cyclodextrin inclusion complex were generated using a USP Type II dissolution apparatus. Complexes equivalent to 25 mg of curcumin were added to simulated gastric fluid (pH 1.2) maintained at 37 ± 0.1°C with a paddle rotation speed of 100 rpm. At predetermined time points, 5-mL aliquots were withdrawn, filtered through Whatman filter paper, and analyzed spectrophotometrically at 423 nm [33].

### 2.3. Formulation Development

#### 2.3.1. Developing a Preformulary Formula

Floating effervescent tablets containing a 1:1 curcumin-methyl-*β*-cyclodextrin complex equivalent to 10 mg curcumin were formulated using a direct compression method. HPMC K15M was incorporated as a sustained release polymer, while microcrystalline cellulose served as a diluent. Sodium bicarbonate and citric acid were included as effervescent agents to impart buoyancy to the tablets. Additionally, talc and magnesium stearate were added as a glidant and lubricant, respectively, to facilitate efficient tablet compression. This formulation strategy is aimed at producing floating matrix tablets capable of controlled curcumin release [34, 35].

#### 2.3.2. Precompression Evaluation

Prior to tablet compression, all formulation ingredients were sieved through an 80-mesh screen, and the required quantities for each batch were carefully weighed. The powder blends were then subjected to a series of tests to evaluate their precompression flow properties. These tests included measurements of bulk density, tapped density, angle of repose, Hausner's ratio, and Carr's compressibility index, providing a comprehensive assessment of powder flow behavior [36, 37].

#### 2.3.3. Preparation of Curcumin Floating Tablets

Floating tablets were formulated to contain a fixed dose of 10 mg curcumin-methyl-*β*-cyclodextrin complex. Microcrystalline cellulose was incorporated as a diluent, while sodium bicarbonate and citric acid served as gas-generating agents to impart buoyancy. A fixed concentration (1% *w*/*w*) of talc and magnesium stearate was included as glidant and lubricant, respectively. To investigate the influence of polymer concentration on drug release, HPMC K15M was incorporated at three different levels: 30%, 40%, and 50% *w*/*w*. The precise quantities of each ingredient were weighed according to the formulation table (Table 1). The powder blend was triturated in a mortar and pestle for 30 min to achieve uniform particle size distribution. Following particle size reduction through a no. 40 sieve, the blend was compressed into tablets using a 16-station tablet punching machine equipped with a 12-mm punch. The tablet weight was kept constant at 420 mg for all formulations [38].

### 2.4. *In Vitro* Buoyancy Characterization of Prepared Curcumin Floating Tablets

#### 2.4.1. Floating Lag Time

To assess the *in vitro* buoyancy, the tablets were placed into a beaker filled with 100 mL of 0.1 N HCl. The time taken for the tablet to rise to the surface and float was recorded as floating lag time (FLT) [39].

#### 2.4.2. Total Floating Time

The duration during which the tablet remained buoyant in 0.1 N HCl was recorded as the total floating time of the tablet [38].

#### 2.4.3. Postcompression Evaluation of Selected F9 Curcumin Floating Tablets

The formulated tablets were rigorously evaluated for key quality parameters, including weight variation, hardness, tablet integrity, thickness, swelling behavior, *in vitro* buoyancy, drug content, and drug dissolution. This comprehensive assessment provided crucial insights into the formulation's characteristics, ensuring that the tablets met the required quality standards and possessed the desired attributes for optimal therapeutic performance.

Tablet hardness was determined using a Monsanto hardness tester. Six tablets were randomly selected from each batch, and their hardness was measured. The average hardness value from five measurements, along with the standard deviation, was reported [29, 40]. Tablet weight uniformity was assessed by randomly selecting 20 tablets from each formulation and weighing them individually using an electronic balance. The mean tablet weight was then calculated [41, 42].

Tablet thickness was determined using a digital Vernier caliper, taking measurements from 20 randomly selected tablets per batch. Friability testing was conducted using a Roche friabilator operated at 25 rpm for 4 min. Then, 10 preweighed tablets from each lot were placed in the friabilator drum and subjected to 100 rotations. After the test, the tablets were dedusted, and the final weight was recorded to calculate the percentage friability [43].

#### 2.4.4. Drug Content (Percentage)

To determine curcumin content, 10 tablets were finely ground, and a weight equivalent to 10 mg of curcumin was accurately transferred to a 10-mL volumetric flask. Then, 5 mL of 0.1 N hydrochloric acid was added, and the mixture was subjected to mechanical shaking for 1 h to ensure complete extraction of the drug. The extract was then filtered through a 0.45-*μ*m filter into a separate 10-mL volumetric flask, and the volume was adjusted with 0.1 N HCl. The absorbance of the resulting solution was measured at 423 nm using a UV-visible spectrophotometer [44].

#### 2.4.5. Swelling Study of Floating Tablets of Curcumin

The swelling behavior of the floating curcumin tablets was evaluated by immersing individual tablets in 100-mL beakers containing 0.1 N HCl. At predetermined time intervals, specifically at each hour, the tablets were carefully extracted from the solution. To ensure accurate measurement of the swelling, excess solution clinging to the tablet surface was gently removed by blotting with tissue paper. This step ensured that only the weight gain due to water uptake by the tablet matrix, and not the weight of the surface liquid, was recorded. Immediately after blotting, the swollen tablets were weighed using an analytical balance, and the weight was documented for each time point.

#### 2.4.6. Drug Release Studies

The release profile of curcumin from the prepared tablet formulations was evaluated using a USP Type II apparatus (paddle method). Dissolution studies were conducted in 900 mL of 0.1 N HCl. The dissolution medium was maintained at a constant temperature of 37°C ± 0.5°C, and the paddle rotated at a speed of 50 rpm. At predetermined time intervals, aliquots of the dissolution medium were withdrawn and immediately replenished with an equal volume of fresh 0.1 N HCl to maintain sink conditions. The collected samples were appropriately diluted, and the absorbance was measured at a wavelength of 423 nm using a Shimadzu 1800 UV-visible spectrophotometer. The cumulative percentage drug release was then calculated from the absorbance values of the samples using the equation derived from the calibration curve [30, 45, 46].

#### 2.4.7. Kinetic Analysis

To gain a comprehensive understanding of the drug release kinetics from formulation F9, dissolution data from six tablets were meticulously analyzed using DDsolver software. This analysis employed a suite of mathematical models, including the Higuchi, Hixson–Crowell, zero-order, first-order, and Korsmeyer–Peppas models, each representing different mechanisms of drug release, such as diffusion, erosion, or a combination thereof. A key parameter derived from the Korsmeyer–Peppas equation, ‘*n*', is determined by plotting the logarithm of percentage drug dissolved against the logarithm of time. This ‘*n*' value provides crucial insights into the dominant release mechanism: An ‘*n*' value of 0.45 signifies Fickian diffusion, while values between 0.45 and 0.89 indicate anomalous transport, suggesting a combination of diffusion and erosion. Values exceeding 0.89 represent Super Case II transport, where erosion of the polymeric chain governs the release. By fitting the dissolution data to these models, the study is aimed at pinpointing the primary mechanism controlling curcumin release from the formulated tablets, ultimately contributing to the optimization of the formulation for achieving the desired therapeutic outcome [28, 47].

## 3. Result and Discussion

### 3.1. Preformulation Studies

The FTIR analysis of curcumin yielded distinct spectral patterns (Table 2). Noteworthy peaks were observed in the curcumin FTIR spectrum. The IR spectrum of the sample shows characteristic peaks for various functional groups. The benzene ring displays a C-C stretching vibration at 1557 cm^−1^. Aromatic compounds exhibit a C=C stretching vibration with an observed peak at 1623.09 cm^−1^. For the ketone functional group, the C=O stretching vibration is noted at 1742.31 cm^−1^. The aromatic C-H stretching vibration is observed at 2927.16 cm^−1^. Phenolic O-H groups are evident with O-H stretching vibrations, having peaks at 3282.26 cm^−1^ and 3222.25 cm^−1^. Additionally, the aromatic C-O stretching vibration is marked by a peak at 1268.25 cm^−1^. Olefinic C-H bending is present with an observed peak at 1428.29 cm^−1^, and the methoxy group is indicated by an O-CH_3_ bending vibration at 1147.31 cm^−1^. All the major absorbance peaks characteristic of the curcumin reference spectra were visible in the spectrum.

The wavelength maxima (*λ*_max_) of curcumin was observed to be 423 nm. The calibration curve of curcumin exhibits a linearity between absorbance and concentration, indicating that the method is suitable for quantifying curcumin within the given range of concentration, specifically 10–70 *μ*g/ml. It demonstrates a robust correlation with an *R*^2^ value of 0.999, signifying a substantial relationship between absorbance and concentration. This implies that the UV-spectrophotometric technique demonstrates reliability in quantifying curcumin within pharmaceutical formulations.

### 3.2. Compatibility of Curcumin With Excipients

The evaluation of drug and excipient interactions was conducted through infrared spectroscopy (IR) analysis of curcumin (Table 2), curcumin-M*β*CD complex (1:1) (Table 2), and curcumin-M*β*CD, HPMC physical mixture (1:1) (Table 2), revealing distinct spectral patterns. No changes were observed in the FTIR spectra of the physical combinations, and all the characteristic absorption wavelengths were undisturbed.

### 3.3. Solubility of Pure Curcumin and Curcumin Cyclodextrin Complex

**Figure 1:**
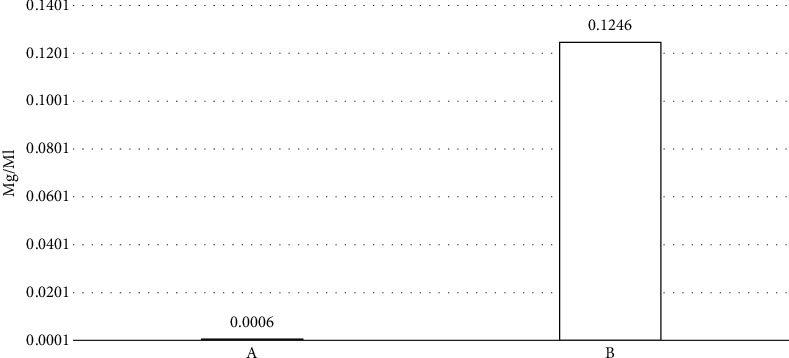
Solubility of pure curcumin and its cyclodextrin complexes: (A) curcumin and (B) curcumin-methyl-*β*-cyclodextrin complexes 1:1.

The assessment of pure curcumin's solubility was conducted in distilled water, with the outcomes 0.0006 mg/mL. The solubility of curcumin-M*β*CD complex (1:1) was 0.12463 mg/mL which was found to be significantly higher than pure curcumin (Figure 1).

### 3.4. *In Vitro* Dissolution of Pure Curcumin and Inclusion Complexes

**Figure 2:**
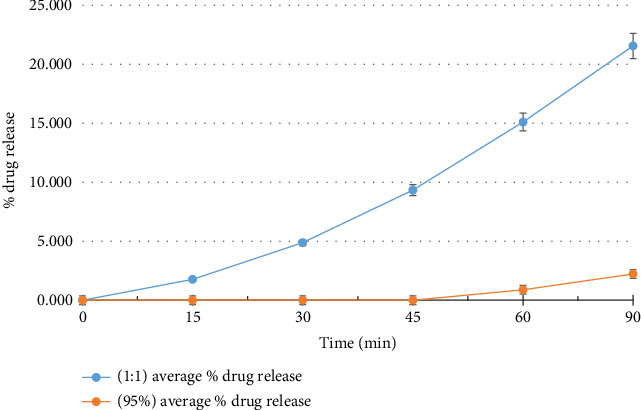
*In vitro* dissolution of curcumin and inclusion complexes (*n*^∗^ = 3).

As depicted in Figure 2, the cyclodextrin inclusion complexes exhibited significantly enhanced dissolution rates compared to pure curcumin during *in vitro* testing. Over the tested duration, the percentage of drug released from pure curcumin was a mere 2.222%, whereas the 1:1 molar ratio M*β*CD-curcumin complexes demonstrated remarkably higher release percentages of 21.556%. This marked improvement in solubility clearly implies that the inclusion complex formulation facilitated faster dissolution of curcumin in comparison to the pure drug.

### 3.5. Formulation Development

Nine different formulations with compositions shown in Table 1 were prepared and evaluated for precompression properties and buoyancy.

### 3.6. Precompression Characterization

Precompression micromeritic characterization was conducted for all the formulations, and the powder mix for F9 exhibited an angle of repose (*θ*) of 27.7578°, a tapped density of 0.7142 g/cm^3^, and a bulk density of 0.6410 g/cm^3^. The computed value of Carr's compressibility index was 10.2492%, indicating moderate compressibility (33, 34), while Hausner's ratio was determined to be 1.114.

### 3.7. *In Vitro* Buoyancy Characteristics of Curcumin Floating Tablets

The floating lag and total floating time exhibited by Formulations 1–9 are detailed in Table 3. Upon contact with the dissolution fluid, the effervescent mixture within the tablets generated carbon dioxide, reducing the tablet density below one and rendering them buoyant.

**Figure 3:**
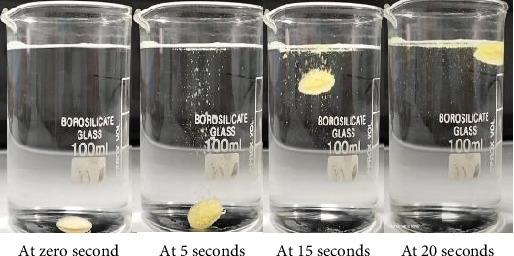
*In vitro* buoyancy of floating tablets of curcumin.

Formulation F9 exhibited an average floating lag of 20.33 ± 4.59 s, as shown in Figure 3, and buoyancy in the 0.1 N HCl, pH 1.2 solution for over 16 h. The higher amount of HPMC K15M allowed for rapid gel layer formation, which trapped the generated CO_2_ efficiently, providing average FLT. Simultaneously, the strong gel matrix maintained tablet integrity, ensuring prolonged FT compared to other formulations with lower polymer or effervescent content. Thus, F9 was selected for further evaluation.

### 3.8. Postcompression Evaluation

The formulation F9 underwent comprehensive quality control assessments in accordance with the standards outlined in the pharmacopeia (39, 41). The tablets exhibited a weight variation of 0.332% ± 1.59%, ensuring uniformity in mass. The thickness of the tablets was consistent, measuring 0.445 ± 0 mm. Friability was found to be 0.2849% ± 0.01%, indicating good mechanical strength and resistance to crumbling. The average weight of the tablets was 421.6 ± 1.64 mg. The tablet hardness was recorded at 5.5 ± 0.07 kg/cm^2^. The prepared tablets were found to be satisfactory in all aspects. The drug content of F9 was determined to be 98.146%, which is well within the acceptable content limit of 90–110%.

### 3.9. Swelling Study of F9 Floating Tablets of Curcumin

The investigation revealed a gradual increase in swelling over time: 24.70% at 1 h, 37.52% at 2 h, and 50.83% at 3 h. Subsequent hours showed continued swelling, with percentages reaching 66.03% at 4 h, 85.98% at 5 h, and finally 98.09% at 6 h. These findings demonstrate the sustained swelling of curcumin floating tablets in acidic conditions which prolongs the floating of tablets and provides a slow release of the drug over the entire period of floatation.

### 3.10. *In Vitro* Drug Release Studies

**Figure 4:**
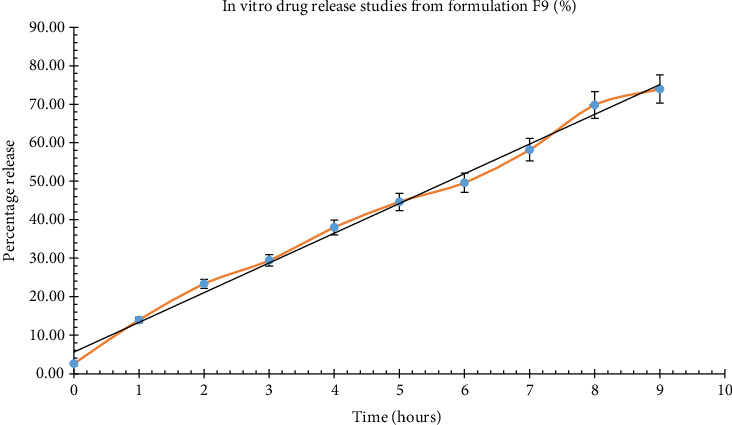
*In vitro* drug release for formulation F9 (⁣^∗^*n* = 6).

Dissolution studies on formulation F9 were carried out in 0.1 N hydrochloric acid (pH 1.2), and the outcomes are illustrated in Figure 4. The preparation showed a drug release of 73.92% over the period of 9 h.

### 3.11. Kinetic Analysis

**Figure 5:**
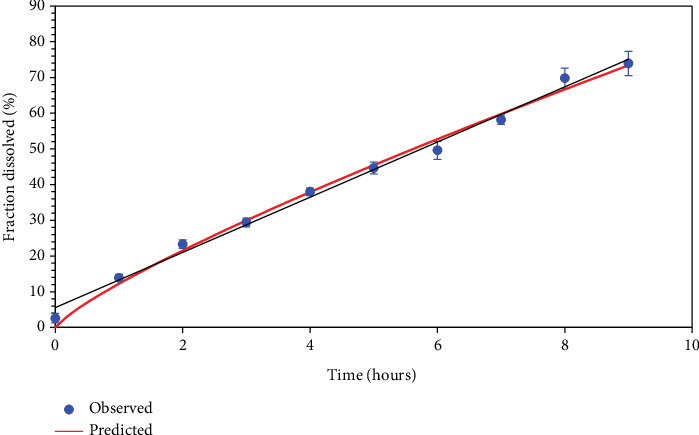
Release kinetics Korsmeyer–Peppas.

The dissolution profiles were analyzed using well-established release kinetic models. The results show that the best-fit model for the data is the Korsmeyer–Peppas model, as indicated by the higher adjusted *R*-squared value of 0.988 and lower, that is, 40.844 Akaike Information Criterion (AIC) in comparison to the first-order model. Additionally, the Korsmeyer–Peppas model yields a higher model selection criterion value (4.425) compared to the first-order model (3.550), further supporting its suitability for describing the release behavior of the tablets. The obtained n value of 0.815 suggests a quasi-Fickian diffusion mechanism. The release kinetics are depicted in Figure 5.

## 4. Conclusion

This innovative formulation presents a promising strategy for improving therapeutic outcomes in the management of peptic ulcers and *H. pylori* infections, addressing significant unmet clinical needs. Curcumin, a natural bioactive compound, offers a favorable safety profile with minimal side effects compared to conventional therapies based on proton pump inhibitors (PPIs) and H_2_ receptor antagonists. The development of floating tablets containing a curcumin–cyclodextrin complex enhances curcumin's aqueous solubility and enables prolonged gastric retention, effectively overcoming its inherent bioavailability limitations. Moreover, this system facilitates site-specific delivery to the stomach, the primary site of action for these indications.

The novelty of this research lies in the formulation of a gastroretentive floating tablet using HPMC K15M, a high-viscosity hydrophilic polymer, to achieve sustained drug release and enhanced oral bioavailability. Unlike traditional delivery systems, this approach leverages a polymer–effervescent combination to optimize both FLT and total floating duration, ensuring extended residence in the stomach. This targeted and sustained delivery platform sets a new direction for curcumin-based therapeutics and represents a significant advancement over existing formulations.

## Figures and Tables

**Table 1:** Composition of floating tablets of curcumin.

**Component (mg)**	**F1**	**F2**	**F3**	**F4**	**F5**	**F6**	**F7**	**F8**	**F9**
Curcumin-methyl-*β*-cyclodextrin complex (1:1)⁣^∗^	20	20	20	20	20	20	20	20	**20**
Microcrystalline cellulose	232	198.4	148	114.4	223.6	156.4	118.6	148	**85**
HPMC K15M	126	126	126	126	126	126	126	168	**210**
Citric acid	8.4	16.8	29.4	37.8	12.6	21	29.4	12.6	**12.6**
Sodium bicarbonate	25.2	50.4	88.2	113.4	29.4	88.2	117.6	63	**84**
Magnesium stearate	4.2	4.2	4.2	4.2	4.2	4.2	4.2	4.2	**4.2**
Talc	4.2	4.2	4.2	4.2	4.2	4.2	4.2	4.2	**4.2**
Total	420	420	420	420	420	420	420	420	**420**

*Note:* The bold entries in the table indicate the best formulation based on the evaluation parameters.

⁣^∗^Each tablet contained a curcumin-M*β*CD inclusion complex (1:1) equivalent to 10 mg of curcumin.

**Table 2:** Fourier transform infrared spectrum analysis of curcumin, curcumin-methyl-*β*-cyclodextrin complex (1:1), and curcumin-methyl-*β*-cyclodextrin complex, hydroxypropyl methylcellulose physical mixture (1:1).

**Functional group**	**Bond vibrations**	**Absorption range (cm** ^**−1**^ **)**	**Observed peaks (cm** ^**−1**^ **)**
	**Curcumin**		
Benzene ring	C-C stretching	1450–1600	1557
Aromatic	C=C stretching	1500–1680	1623.09
Ketone	C=O stretching	1670–1750	1742.31
Aromatic	C-H stretching	2920–3079	2927.16
Phenolic O-H	O-H stretching	3200–3500	3282.26 and 3222.25
Aromatic	C-O stretching	1000–1300	1268.25
Olefin	C-H bending	1400–1500	1428.29
Methoxy	O-CH_3_ bending	1050–1100	1147.31
	**Methyl-** *β * **-cyclodextrin**		
Hydroxyl-OH	O-H stretching	3200–3550	3400
Alkane	C-H stretching	2850–2960	2920, 2850
Carbonyl	C=O stretching	1700–1748	1742.31
Ether	C-O stretching	1050–1150	1080
	**HPMC**		
Hydroxyl-OH	O-H stretching	3200–3550	3440
Alkane	C-H stretching	2850–2960	2930
Ether	C-O stretching	1050–1150	1100
Methyl	C-H Bend	1375–1450	1380

**Table 3:** Experimental data: buoyancy of curcumin floating tablets.

**Formulations**	**Floating lag time**	**Floating time**
F1	Immersed	Immersed
F2	13.17 ± 1.60 min	1.0 ± 0.00 h
F3	20.67 ± 2.73 min	0.2 ± 0.00 h
F4	29.67 ± 4.68 min	0.1 ± 0.01 h
F5	1.67 ± 0.82 min	1.0 ± 0.00 h
F6	3.83 ± 1.47 min	0.5 ± 0.00 h
F7	4.83 ± 2.32 min	0.5 ± 0.00 h
F8	11.50 ± 3.02 min	5.5 ± 0.00 h
F9	20.33 ± 4.59 s	16 ± 0.10** h**

*Note:* The bold entries in the table indicate the best formulation based on the evaluation parameters.

⁣^∗^*N* = 6, and each reading is shown as average ± standard deviation.

## Data Availability

Data used in this study is available within the manuscript.
